# Placental mTOR complex 1 regulates fetal programming of obesity and insulin resistance in mice

**DOI:** 10.1172/jci.insight.149271

**Published:** 2021-07-08

**Authors:** Brian Akhaphong, Daniel C. Baumann, Megan Beetch, Amber D. Lockridge, Seokwon Jo, Alicia Wong, Tate Zemanovic, Ramkumar Mohan, Danica L. Fondevilla, Michelle Sia, Maria Ruth B. Pineda-Cortel, Emilyn U. Alejandro

**Affiliations:** 1 Department of Integrative Biology & Physiology, University of Minnesota Medical School, Minneapolis, Minnesota, USA.; 2 Research Center for the Natural and Applied Sciences and; 3 Department of Medical Technology, University of Santo Tomas, Manila, Philippines.

**Keywords:** Endocrinology, Metabolism, Diabetes, Islet cells, Obesity

## Abstract

Fetal growth restriction, or low birth weight, is a strong determinant for eventual obesity and type 2 diabetes. Clinical studies suggest placental mechanistic target of rapamycin (mTOR) signaling regulates fetal birth weight and the metabolic health trajectory of the offspring. In the current study, we used a genetic model with loss of placental mTOR function (mTOR-KO^Placenta^) to test the direct role of mTOR signaling on birth weight and metabolic health in the adult offspring. mTOR-KO^Placenta^ animals displayed reduced placental area and total weight, as well as fetal body weight at embryonic day (E) 17.5. Birth weight and serum insulin levels were reduced; however, β cell mass was normal in mTOR-KO^Placenta^ newborns. Adult mTOR-KO^Placenta^ offspring, under a metabolic high-fat challenge, displayed exacerbated obesity and metabolic dysfunction compared with littermate controls. Subsequently, we tested whether enhancing placental mTOR complex 1 (mTORC1) signaling, via genetic ablation of TSC2, in utero would improve glucose homeostasis in the offspring. Indeed, increased placental mTORC1 conferred protection from diet-induced obesity in the offspring. In conclusion, placental mTORC1 serves as a mechanistic link between placental function and programming of obesity and insulin resistance in the adult offspring.

## Graphical abstract

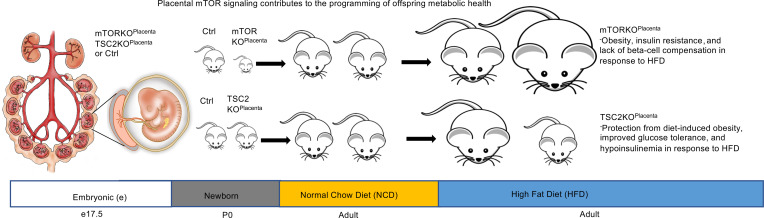

## Introduction

Type 2 diabetes (T2D) affects more than 350 million individuals worldwide (1). It is a complex disease characterized by pancreatic β cell failure in the setting of obesity and insulin resistance in the peripheral tissues. Known genetic variants account for less than 10% of the risk for T2D (2, 3). Evidence from human epidemiology as well as animal studies shows that fetal nutrient environmental factors provide significant susceptibility to T2D (2–6). Indeed, adverse maternal influences in pregnancy are linked to alteration in fetal birth weight and the long-term health of the offspring. In the United States, about 8% of infants are born with low birth weight (7), a proxy for poor fetal growth. Placental insufficiency is the primary cause of fetal growth restriction (FGR) or low birth weight, which are strong determinants for eventual development of obesity and T2D (8).

The placenta senses and responds to changes in the maternal environment by altering its structure and function (e.g., nutrient transport) and matching maternal resources and fetal growth (9). The placenta is thought to integrate maternal signals to placental function through the mechanistic target of rapamycin (mTOR) kinase. mTOR transduces signals from nutrients and growth factors to regulate cellular growth and organ size, but its role in the placenta remains poorly understood. mTOR is highly expressed in the syncytiotrophoblast of the human placenta (10), and its expression and activity are regulated by glucose, amino acids (AAs), and hormones such as insulin and IGF-1. Association-based human studies suggest that fetal placental mTOR signaling may serve as a critical link between the maternal nutrient supply and growth of the developing fetus (11). Possible mechanisms by which mTOR can regulate fetal growth may involve regulation of nutrient transporters in the placenta (10, 12) and trophoblast cells’ development (13). FGR in human and animal models show reduced concentrations of placental AAs, such as leucine, that are associated with reduced mTOR activity (10, 14–16). Downstream of mTOR, the mTOR complex 1 (mTORC1) pathway is negatively regulated by TSC2. mTORC1 is known to affect placental AA transporter activity (16), while mTORC2/Akt signaling regulates trophoblast development (13). Therefore, placental mTOR signaling may not only direct placental metabolism and growth but also influence the development and long-term health trajectory of the offspring. However, the role of placental mTOR signaling in fetal programming of metabolic disease has not been directly studied in vivo.

Human studies show that reduced placental mTOR activity is correlated with FGR (10, 17), whereas increased activity is positively correlated with fetal overgrowth, such as in maternal obesity (18). Fetal overgrowth, or macrosomia, is an adverse outcome of gestational diabetes mellitus (GDM) and increases the risk of the offspring of developing obesity/T2D in adulthood (19). A causal relationship between placental mTOR and fetal birth weight and long-term health of the offspring has not been established. Thus, a murine model testing the direct role of placental mTOR on fetal programming of metabolic health of the offspring would fill this knowledge gap.

In the present study, we tested the hypothesis that placental mTOR regulates fetal birth weight as well as the development of obesity and T2D in the adult offspring. We used the specific placental CYP19Cre recombinase to genetically delete mTOR or to increase mTORC1 activity through the deletion of TSC2, the negative regulator of mTORC1, in trophoblast cells of the placenta. Characterization of the metabolic phenotypes in the offspring at birth and in adulthood, under normal and high-fat diet (HFD) conditions, revealed an increased susceptibility to obesity and glucose homeostasis dysfunction in the adult offspring that experienced loss of placental mTOR (*CYP19Cre^+^ mTOR^fl/fl^*, hereafter mTOR-KO^Placenta^), whereas gain of placental mTORC1 activity conferred protection under metabolic stress. Our data demonstrate direct evidence for placental mTOR signaling in the regulation of fetal growth and the programming of obesity and T2D. These studies provide a strong rationale to target placental mTOR signaling in FGR-related complications in human pregnancy.

## Results

### Generation of genetic placental mTOR ablation model in mice.

**Figure 1:**
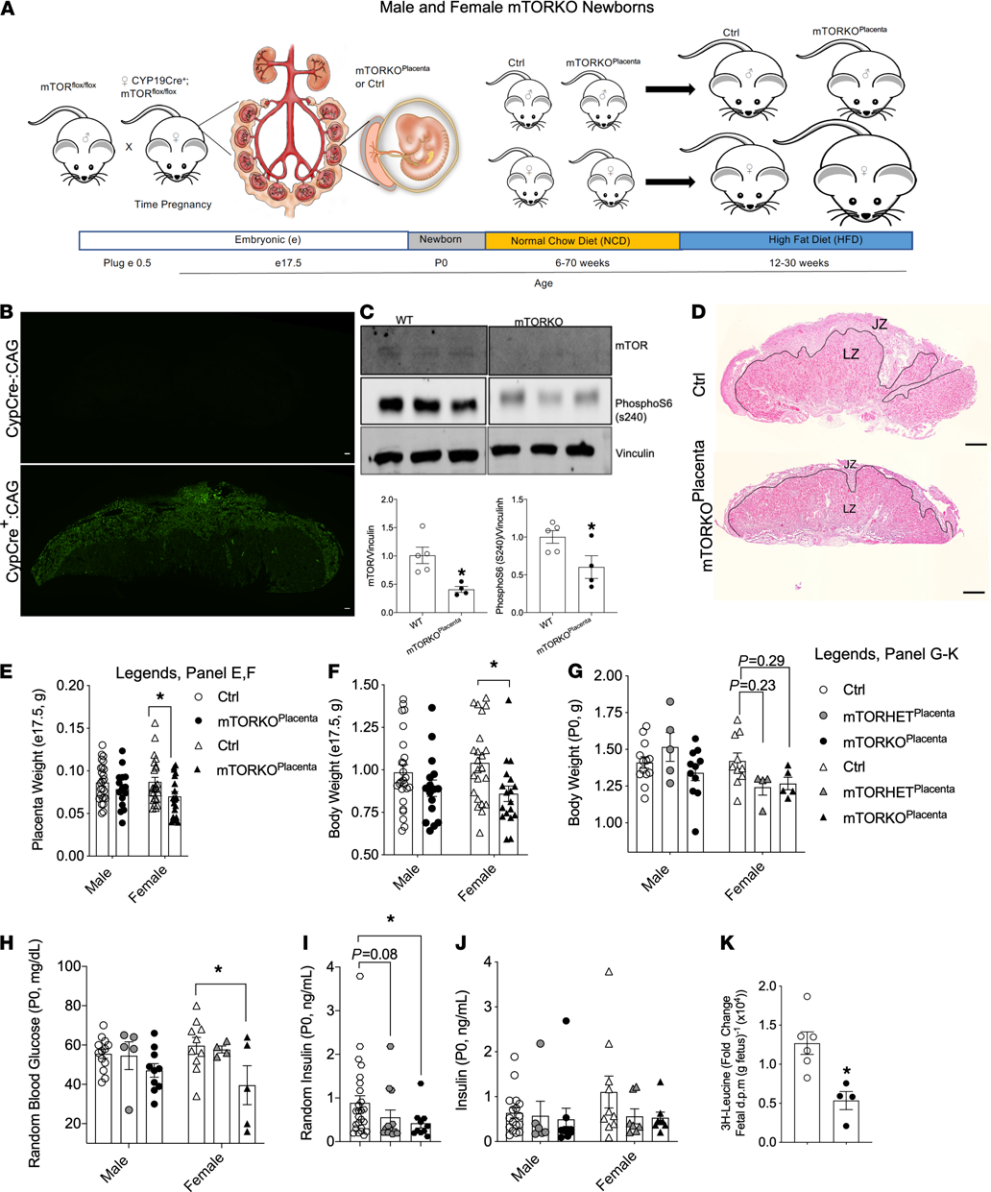
Reduced placental weight and fetal body weight in mTOR-KO^Placenta^ embryos. Schematic diagram and timeline of the mTOR-KO study (**A**). Endogenous GFP reporter (CAG-ZsGreen1) in control (*CYP19Cre^–^*), positive control (*CYP19Cre^+^*) experimental placentas (**B**). Representative Western blot of E15.5 placenta lysates for mTOR and phosphorylated S6 (Ser 240) and respective quantifications normalized to vinculin (**C**). H&E staining of control (Ctrl) and mTOR-KO^Placenta^ (**D**, junctional zone [JZ] and labyrinth zone [LZ]). Placental weight of E17.5 males (*n* = 25 Ctrl, 16 mTOR-KO^Placenta^) and females (*n* = 24 Ctrl, 18 mTOR-KO^Placenta^, **E**). E17.5 fetal weight separated by male (*n* = 25 Ctrl, 16 mTOR-KO^Placenta^) and female (*n* = 23 Ctrl, 18 mTOR-KO^Placenta^, **F**). P0 (within 12 hours of birth) body weight separated by male (*n* = 13 Ctrl, 5 mTOR-HET^Placenta^, 11 mTOR-KO^Placenta^) and female (*n* = 10 Ctrl, 4 mTOR-HET^Placenta^, 5 mTOR-KO^Placenta^, **G**). Random blood glucose was measured from trunk blood of P0 males (*n* = 13 Ctrl, 5 mTOR-HET^Placenta^, 10 mTOR-KO^Placenta^) and females (*n* = 10 Ctrl, 4 mTOR-HET^Placenta^, 5 mTOR-KO^Placenta^, **H**). Random insulin serum was measured from trunk blood of P0 Ctrl, mTOR-HET^Placenta^, and mTOR-KO^Placenta^ pups (*n* = 25, 13, 10, respectively) from 5 dams (**I**). Random insulin serum was measured from trunk blood of P0 Ctrl, mTOR-HET^Placenta^, and mTOR-KO^Placenta^ males (*n* = 17, 6, 10, respectively) and females (*n* = 10, 7, 9, respectively) from 5 dams (**J**). Fetal ^3^H-Leucine uptake after 10 minutes of leucine placental perfusion in utero (*n* = 4–6) from 3 dams (**K**). Statistical analysis was performed using 2-tailed Mann-Whitney (**C**, **I**, and **K**) and 2-way ANOVA with Sidak’s multiple comparisons (**E**–**G**, **H**, and **J**). Error bars represent mean ± SEM. *P < 0.05 Ctrl vs. mTOR-KO^Placenta^. Scale bars in images (**B** and **D**) are 200 μm.

To understand the impact of placental mTOR on fetal weight and metabolic health, we created a mouse model in which mTOR is genetically deleted in the placenta by utilizing the human CYP19 gene to drive the Cre recombinase transgene starting at embryonic day (E) 6.5 (20), a technique previously shown to effectively induce placenta-specific gene deletion in the mouse (21, 22). The schematic diagram of the study is shown in Figure 1A. First, we verified placental specificity of the CYP19Cre using a green fluorescent protein reporter line (CAG-GFP, Figure 1B), where we detected GFP throughout the entire placenta only in CYP19Cre E17.5 embryos. By Western blot, we determined a significant reduction in mTOR protein level, as well as the phosphorylation status of S6 (Ser 240), a downstream target of mTOR, in E15.5 placenta lysates of mTOR-KO^Placenta^ compared with WT controls (Figure 1C). X-CLARITY–based tissue clearing and spectral imaging in whole bodies of newborns (postnatal day 0, P0) showed relatively equal low GFP background signal between mTOR-KO^Placenta^ and WT Ctrl (GFP^–^, Supplemental Figure 1A; supplemental material available online with this article; https://doi.org/10.1172/jci.insight.149271DS1), suggesting lack of reporter expression in the whole body of both genotypes (Ctrl and *CYP19Cre^+^ CAG-GFP*) (22). Supporting the X-CLARITY data, we observed comparable levels of mTOR protein in whole embryonic livers, in addition to visceral fat and islets in adult mTOR-KO^Placenta^ and Ctrl offspring (Supplemental Figure 1, B–D).

### Reduced placental weight and fetal body weight in mTOR-KO^Placenta^ offspring.

In human pregnancies, decreased mTOR signaling is associated with decreased birth weight (10). To better understand the impact of placental mTOR loss on embryonic growth, we assessed the weight and architecture of the placenta on E17.5. Representative H&E images of E17.5 placenta are shown in Figure 1D. Consistent with the known association of placental mTOR signaling and fetal growth, we observed a reduction in placental area (Supplemental Figure 2A). Total placental weight was reduced in mTOR-KO^Placenta^ animals at E17.5 (Supplemental Figure 2B), and this was significant in female mTOR-KO^Placenta^ animals (Figure 1E). The LZ mass was smaller, and a trend toward decrease in JZ mass was also observed in E17.5 mTOR-KO^Placenta^ (*P* = 0.07, Supplemental Figure 2, C and D). To control body weight measurement variability in P0 (due to differences in time of delivery), we measured body weight at E17.5. A significant reduction in body weight was observed in E17.5 and P0 mTOR-KO^Placenta^ (Supplemental Figure 2E). However, significance was observed only in females at E17.5 mTOR-KO^Placenta^ (Figure 1, F and G). Together, these data show that deletion of placental mTOR reduced body weight of the offspring.

### Normal pancreatic β cell mass but reduced serum insulin levels in mTOR-KO^Placenta^ offspring.

Pancreas development is sensitive to nutrient availability in utero (4, 23). Female mTOR-KO^Placenta^ mice displayed reduced nonfasted blood glucose (Figure 1H and Supplemental Figure 2F). Nonfasted serum insulin levels were reduced in mTOR-KO^Placenta^ in P0 animals (Figure 1I). After considering sex as a variable, there were no significant changes observed between groups (Figure 1J). Normal β cell mass was detected in both male and female mTOR-KO^Placenta^ mice compared with littermate controls (Supplemental Figure 2, G and H). Collectively, these data show that deletion of placental mTOR reduced insulin levels and decreased glucose, but not β cell mass, in the newborn offspring.

### Reduced leucine transfer from mother to mTOR-KO^Placenta^ offspring.

Placental mTOR has been previously implicated to regulate AA transfer from mother to fetus (24, 25). Next we measured plasma AAs in P0 mTOR-KO^Placenta^ and littermate controls, but we found no differences among all the AAs detected (Supplemental Figure 3A). Since mTORC1 has been shown to regulate system A and system L AA transport in primary human trophoblast cells (12), and leucine transport is downregulated in FGR (10), we specifically measured leucine transfer from the maternal to fetal circulation with or without placental mTOR deletion. Our data showed reduced leucine transport from mothers to embryos of mTOR-KO^Placenta^ compared with littermate controls (Figure 1K).

### Adult male and female mTOR-KO^Placenta^ offspring display normal glucose homeostasis phenotypes.

Low birth weight is a strong determinant for eventual metabolic dysfunction in adulthood. To assess the metabolic impact of placental mTOR in adult offspring, we followed offspring from birth to adulthood and performed in vivo phenotyping under normal chow diet (NCD). Fetal programming can have sexually dimorphic effects in the offspring; therefore, metabolic phenotyping was done in both sexes. Male mTOR-KO^Placenta^ mice displayed a mildly increased body weight starting at week 10 (Supplemental Figure 4A) compared with their littermate controls. Nonfasted blood glucose was normal at 11–20 weeks of age (Supplemental Figure 4B). At 8 weeks of age, male mTOR-KO^Placenta^ mice displayed normal glucose tolerance (Supplemental Figure 4C) and insulin sensitivity (Supplemental Figure 4D). Comparable body weight (Supplemental Figure 4E) and nonfasted blood glucose at 11–20 weeks of age were observed in female mTOR-KO^Placenta^ compared with littermate controls (Supplemental Figure 4F). Similarly, female mTOR-KO^Placenta^ mice displayed normal glucose tolerance and insulin sensitivity at 12 weeks (Supplemental Figure 4, G and H).

### Adult female mTOR-KO^Placenta^ mice display exacerbated obesity phenotypes under HFD challenge.

**Figure 2:**
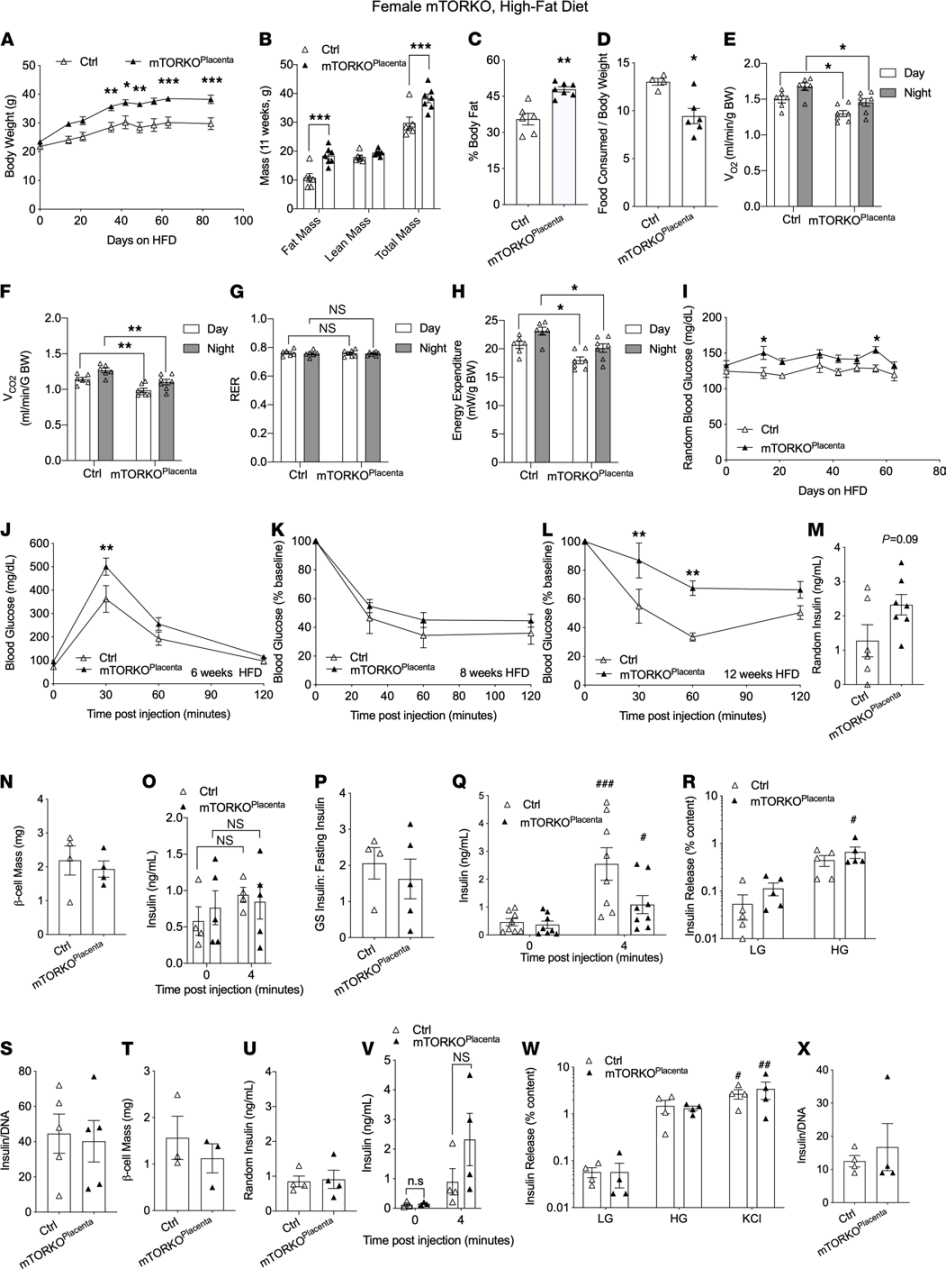
mTOR-KO^Placenta^ females become obese, glucose intolerant, and insulin resistant after HFD treatment. Weight of Ctrl versus mTOR-KO^Placenta^ over 90 days of HFD starting at 17 weeks of age (*n* = 6, 7, respectively; **A**). EchoMRI body composition measurements after 11 weeks of HFD (*n* = 6, 7, **B**) and body fat percentage (**C**). Food consumed per gram body weight over 48 hours (*n* = 4, 6, **D**). VO_2_ of Ctrl versus mTOR-KO^Placenta^ at 11 weeks of HFD (*n* = 6, **E**). VCO_2_, RER, and energy expenditure (*n* = 6, **F**–**H**). Random blood glucose of HFD (*n* = 6, 7, **I**). Intraperitoneal glucose tolerance test (IPGTT) after 16 hours of fasting on HFD at 6 weeks (*n* = 6, 7, **J**). Insulin tolerance test (ITT) after 6 hours of fasting at 8 weeks of HFD (*n* = 6, 7, **K**). ITT after 6 hours of fasting at 12 weeks of HFD (*n* = 6, 7, **L**). Random serum insulin at 10 weeks of HFD (*n* = 6, 7, **M**). β Cell mass at 13 weeks of HFD (*n* = 4 each group, **N**). In vivo GSIS and secretory ratio at 10 weeks of HFD (*n* = 4 Ctrl, 5 mTOR-KO^Placenta^, **O** and **P**). In vivo GSIS on NCD, 12 weeks old (*n* = 8 each group, **Q**). In vitro GSIS on normal chow, 12 weeks old (*n* = 5 each group, **R**), and islet insulin content (*n* = 5 each group, **S**). β Cell mass at 12 weeks old (*n* = 3 Ctrl, 3 mTOR-KO^Placenta^, **T**). Random insulin serum of normal chow at 70 weeks old (*n* = 4 each group, **U**). In vivo and in vitro GSIS of normal chow 70-week-old mice (*n* = 4 each group, **V** and **W**). Islet insulin content of normal chow 70-week-old mice (*n* = 4 each group, **X**). LG, low glucose; HG, high glucose. Statistical analysis was performed using 2-tailed Mann-Whitney (**C**, **D**, **M**, **N**, **P**, **S**–**U**, and **X**) and 2-way ANOVA Sidak’s multiple comparisons (**A**, **B**, **E**–**L**, **O**, **Q**, **R**, **V**, and **W**) with repeated measures when appropriate. Error bars represent mean ± SEM. *P < 0.05, **P < 0.01, ***P < 0.001, Ctrl vs. mTOR-KO^Placenta^. ^#^*P* < 0.05, ^##^*P* < 0.01, ^###^*P* < 0.001 vs. time point 0 or LG (within genotype).

Sex differences are observed in animal models of fetal programming (26). Therefore, we tested whether female mTOR-KO^Placenta^ mice are more susceptible to a “second hit” insult, such as HFD. Assessment of body weight throughout the course of HFD treatment revealed significant weight gain in the female mTOR-KO^Placenta^ compared with littermate controls starting after 40 days of HFD (Figure 2A). Fat mass via EchoMRI body composition analysis (Figure 2B) was increased in female mTOR-KO^Placenta^ (Figure 2C) after 11 weeks of HFD. Increased food intake can contribute to obesity. However, female mTOR-KO^Placenta^ mice ate less relative to their body weight compared with controls (Figure 2D). We assessed whether energy expenditure could account for the obesity phenotype in female mTOR-KO^Placenta^ mice. Basal O_2_ and CO_2_ (VO_2_ and VCO_2_) were decreased (Figure 2, E and F), and normal respiratory exchange ratio (RER) during the day and night were observed in mTOR-KO^Placenta^ compared with littermate controls (Figure 2G). Energy expenditure was also reduced in mTOR-KO^Placenta^ females during both day and night cycles compared with controls (Figure 2H). Nonfasted hyperglycemia was also evident in mTOR-KO^Placenta^ compared with littermate controls (Figure 2I).

### Adult female mTOR-KO^Placenta^ mice display glucose and insulin intolerance and show deficiency in β cell mass compensation under HFD challenge.

After 6 weeks of HFD, female mTOR-KO^Placenta^ displayed glucose intolerance (Figure 2J) but normal insulin sensitivity at 8 weeks of HFD treatment (Figure 2K). However, a significant insulin resistance (Figure 2L) was observed at 12 weeks of HFD in mTOR-KO^Placenta^ compared with littermate controls. A trend toward hyperinsulinemia at 10 weeks of HFD was observed in mTOR-KO^Placenta^ mice (*P* = 0.09, Figure 2M). At 13 weeks of HFD β cell mass between mTOR-KO^Placenta^ and controls was normal (Figure 2N). Next we tested for β cell function via glucose-stimulated insulin secretion (GSIS) and found that the mTOR-KO^Placenta^ and littermate control mice under HFD displayed similar serum insulin levels in response to high-glucose treatment (Figure 2O), and the insulin secretion stimulation index was the same (Figure 2P) under HFD treatment. Together these data suggest that mTOR-KO^Placenta^ mice have increased susceptibility to glucose homeostasis dysfunction, in part, due to lack of β cell mass compensation for insulin resistance.

### Adult female mTOR-KO^Placenta^ mice display transient reduction in β cell function.

To remove the confounding effects of HFD treatment, we assessed basal β cell function in mTOR-KO^Placenta^ and control female mice under NCD. In the GSIS experiment, serum insulin was reduced in mTOR-KO^Placenta^ (Figure 2Q). However, at the islet level, in vitro GSIS in 12-week-old mice under normal chow displayed normal response to high glucose (Figure 2R). Islet insulin content (Figure 2S) and β cell mass were found to be comparable between mTOR-KO^Placenta^ and controls at 12 weeks of age (Figure 2T). Additionally, β cell function was assessed in older mice (70 weeks). Nonfasted serum insulin levels were not different between groups (Figure 2U). GSIS responses in vivo or in vitro (Figure 2, V and W) between mTOR-KO^Placenta^ and littermate controls were comparable. Moreover, islet insulin content at this age was also normal (Figure 2X). These data suggest that the reduced serum insulin level was transient and point to the defect in extraislet tissues.

### Normal placental weight and fetal body weight in E17.5 embryos with gain of placental mTORC1.

**Figure 3:**
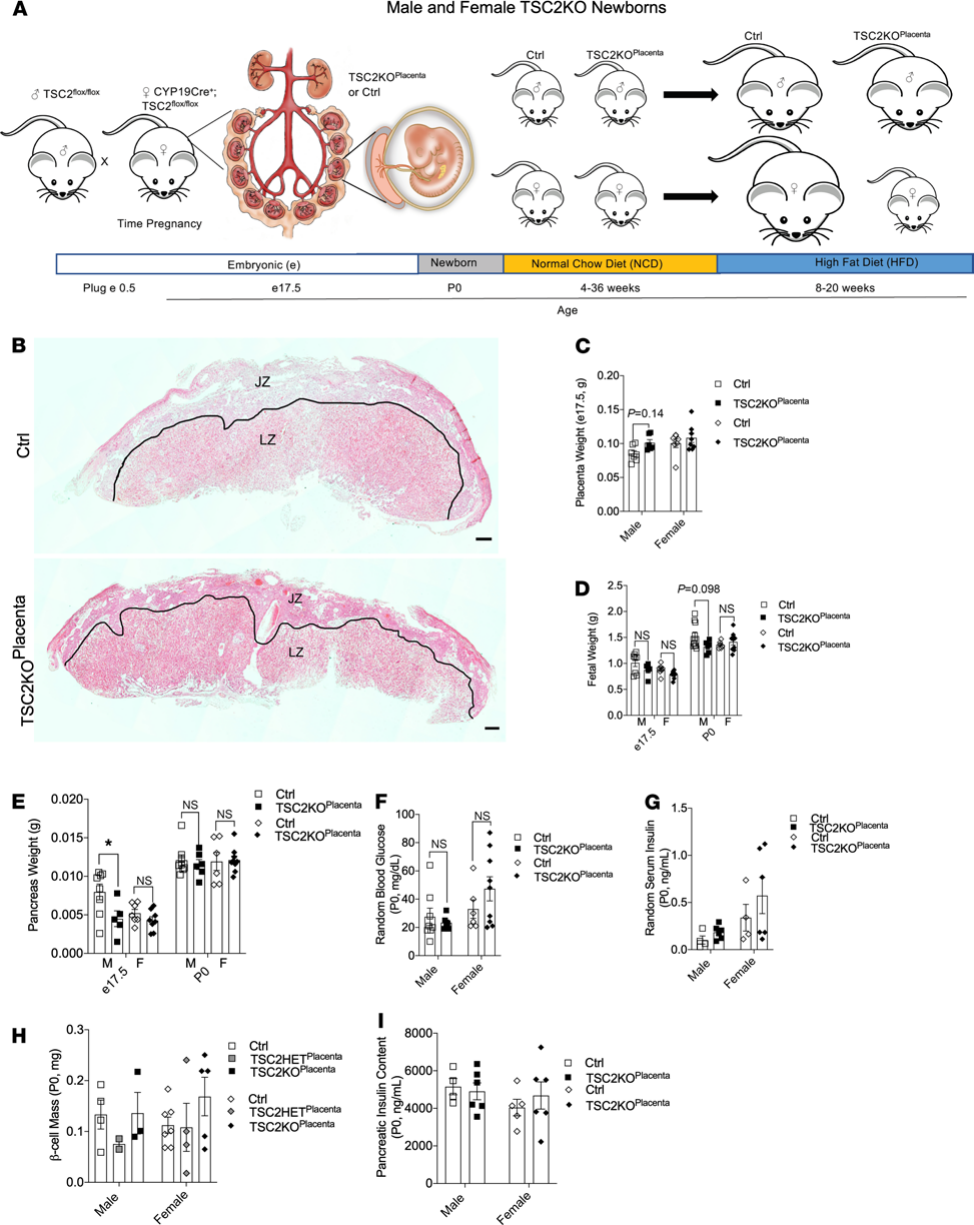
TSC2-KO^Placenta^ newborn offspring have normal placental weight and β cell mass. Schematic diagram and timeline of the TSC2-KO study (**A**). H&E staining of E17.5 Ctrl and TSC2-KO^Placenta^ (**B**). Placental weights of male Ctrl versus TSC2-KO^Placenta^ (*n* = 7, 6) and female Ctrl versus TSC2-KO^Placenta^ (*n* = 7, 8) mice at E17.5 (**C**). Fetal weights of male (*n*_E17.5_ = 8, 5; *n*_P0_ = 10, 6) and female (*n*_E17.5_ = 7, 8; *n*_P0_ = 6, 9) Ctrl vs. TSC2-KO^Placenta^ mice at E17.5 and P0 (**D**). Pancreas weights of male (*n*_E17.5_ = 8, 5; *n*_P0_ = 8, 6) and female (*n*_E17.5_ = 7, 8; *n*_P0_ = 6, 9) mice measured at E17.5 and P0 (**E**). Random blood glucose of males (*n* = 8, 6) and females (*n* = 6, 9) at P0 (**F**). Random serum insulin of males (*n* = 4, 6) and females (*n* = 4, 6) at P0 (**G**). β Cell mass of males (*n* = 4 Ctrl, 2 TSC2-HET^Placenta^, 3 TSC2-KO^Placenta^) and females at P0 (*n* = 7 Ctrl, 4 TSC2-HET^Placenta^, 5 TSC2-KO^Placenta^, **H**). Whole pancreatic insulin content of males (*n* = 4 Ctrl, 6 TSC2-KO^Placenta^) and females (*n* = 5 Ctrl, 6 TSC2-KO^Placenta^) at P0 (**I**). Statistical analysis was performed using 2-way ANOVA with Sidak’s multiple comparisons (**C**–**I**). Error bars represent mean ± SEM. Scale bars in images are 500 μm. *P < 0.05 Ctrl vs. TSC2-KO^Placenta^.

Similar to human studies showing association of decreased mTOR with FGR (17), the mTOR-KO^Placenta^ shows decreased fetal weight. However, the impact of increased placental mTOR signaling has not been studied. We hypothesized that increased mTORC1 would be associated with increased fetal weight. We tested this directly by genetically deleting TSC2, the negative regulator of mTORC1 (*CYP19Cre^+^ TSC2^fl/fl^*, hereafter TSC2-KO^Placenta^; Figure 3A). Representative images of placentas from TSC2-KO^Placenta^ and littermate controls are shown in Figure 3B. The JZ and LZ masses of TSC2-KO^Placenta^ were not changed in either sex (Figure 3B and Supplemental Figure 5, A–D). Male and female TSC2-KO^Placenta^ placental weights at E17.5 were comparable (Figure 3C). At E17.5 and P0, the fetal weight was comparable between groups (Figure 3D). The pancreas weight was decreased in male TSC2-KO^Placenta^ at E17.5, whereas female pancreatic weight was unchanged at E17.5 and P0 (Figure 3E). Nonfasted blood glucose, insulin, β cell mass, and pancreas insulin content were comparable between genotypes at P0 (Figure 3, F–I). Together these data show that increased mTORC1 signaling did not alter placental weight, body weight, or β cell mass in the offspring. Although these results in neonatal pancreas were unexpected, the increased placental mTOR may still have long-term metabolic consequences in the adult offspring.

### Adult male and female TSC2-KO^Placenta^ mice show improved insulin sensitivity under NCD.

To assess the impact of enhanced mTORC1 signaling during fetal development in adult offspring, we investigated glucose homeostasis status under standard diet. No difference in body weight was observed between male TSC2-KO^Placenta^ and littermate controls (Supplemental Figure 6A). Fasting but not nonfasting blood glucose was significantly decreased between 8-week-old male TSC2-KO^Placenta^ and littermate controls (Supplemental Figure 6B). No difference in glucose tolerance was detected in 6- to 20-week-old males (Supplemental Figure 6C). However, improved insulin sensitivity was evident in 7- to 12-week-old male TSC2-KO^Placenta^ compared with littermate controls (Supplemental Figure 6D). In vitro β cell function, measured by insulin secretion in response to high glucose, KCl, and palmitate, was comparable between primary isolated islets from control and TSC2-KO^Placenta^ mice (Supplemental Figure 6E). Total islet insulin content (Supplemental Figure 6F) and β cell mass were comparable between genotypes (Supplemental Figure 6G). The female TSC2-KO^Placenta^ and littermate controls demonstrated comparable body weight gain in adulthood (Supplemental Figure 6H). No differences were detected in fasting or nonfasting blood glucose in mice at 12–20 weeks of age (Supplemental Figure 6I). Adult female TSC2-KO^Placenta^ mice demonstrated normal glucose tolerance (Supplemental Figure 6J) and insulin sensitivity (AUC *P* = 0.14, Supplemental Figure 6K).

### Adult male TSC2-KO^Placenta^ mice display improved glucose tolerance in HFD challenge.

**Figure 4:**
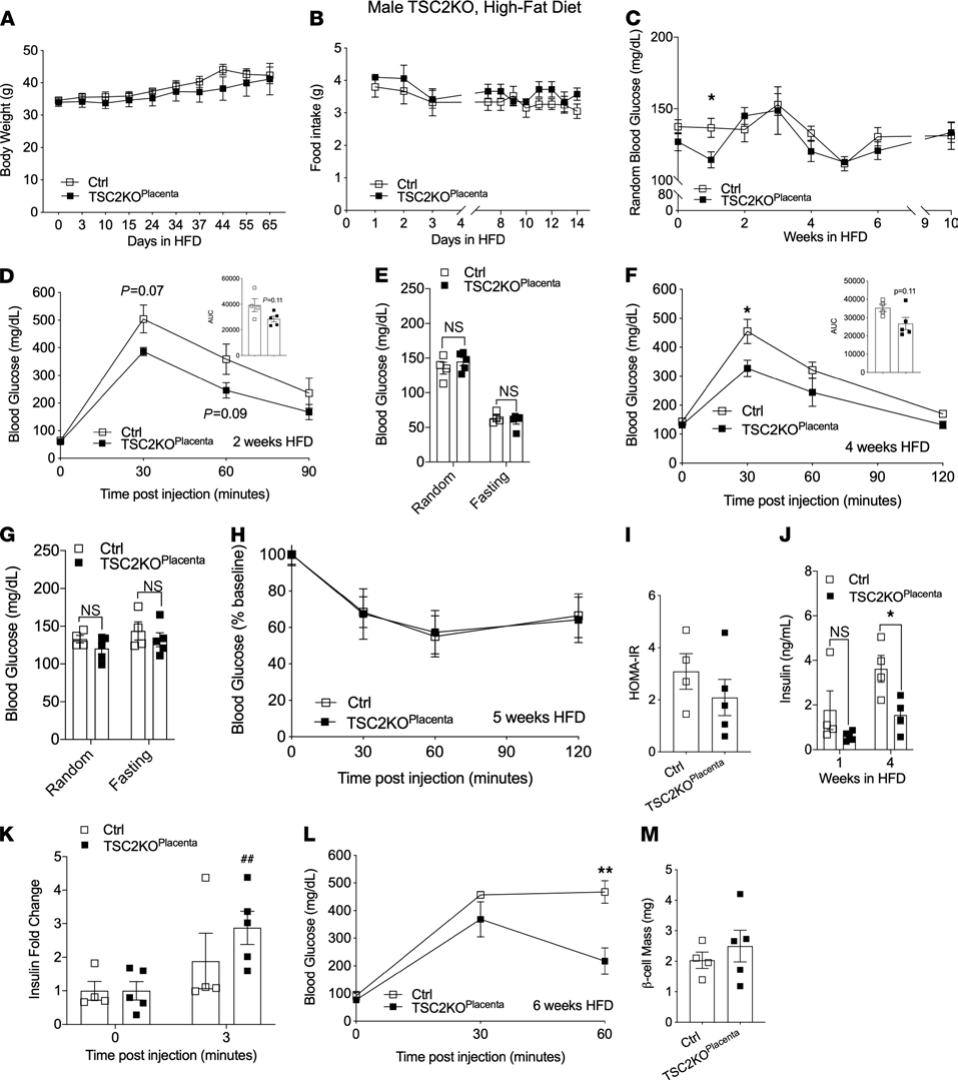
Adult male TSC2-KO^Placenta^ mice are protected from obesity-induced metabolic dysfunction. Weight progression of Ctrl versus TSC2-KO^Placenta^ male mice fed with HFD over 9 weeks starting at 17 weeks of age (*n* = 4, 5, respectively, **A**). Food intake measurements of HFD male mice (*n* = 4, 5, **B**). Random blood glucose of HFD male mice (*n* = 4, 5, **C**). IPGTT after 16 hours of fasting at 2 weeks HFD (*n* = 4, 5, **D**) and corresponding random and fasting blood glucose (*n* = 4, 5, **E**). IPGTT after 16 hours of fasting at 4 weeks of HFD (*n* = 4, 5, **F**) in addition to random and fasting blood glucose (*n* = 4, 5, **G**). ITT at 5 weeks of HFD after 6 hours of fasting (*n* = 4, 5, **H**). HOMA-IR of HFD males at 6 weeks (*n* = 4, 5, **I**). Random insulin serum levels at 1 and 4 weeks of HFD (*n* = 4, 4–5, **J**). In vivo GSIS on males at 6 weeks of HFD (*n* = 4, 5, **K**) and concurrent IPGTT (*n* = 4, 5, **L**). β Cell mass of males at 10 weeks of HFD (*n* = 4, 5, **M**). Statistical analysis was performed using 2-tailed Mann-Whitney (**I** and **M**) and 2-way ANOVA with Sidak’s multiple comparisons (**A**–**H** and **J**–**L**) with repeated measures when appropriate. Error bars represent mean ± SEM. *P < 0.05, **P < 0.01 Ctrl vs. TSC2-KO^Placenta^. ^##^*P* < 0.01 vs. time point 0 (within genotype).

To test that enhanced mTORC1 is sufficient to improve metabolic outcomes in high-nutrient stress exposure, adult male TSC2-KO^Placenta^ and littermate controls were put on an HFD for 10 weeks. Throughout the HFD, the male TSC2-KO^Placenta^ and littermate controls showed comparable body weights (Figure 4A) and food intake (Figure 4B). Nonfasted blood glucose levels were similar throughout the HFD feeding, with an exception at week 1 (Figure 4C). A trend toward improved glucose tolerance was observed in male TSC2-KO^Placenta^ compared with littermate controls after 2 weeks of HFD (*P* = 0.07, Figure 4D), suggesting a protective effect of placental mTORC1 on the adult offspring. At 2 weeks of HFD, nonfasted or fasting blood glucose levels were comparable between the groups (Figure 4E). At 4 weeks of HFD, improved glucose tolerance persisted in TSC2-KO^Placenta^ compared with littermate controls (Figure 4F). At this point of HFD, no difference in nonfasting or fasting blood glucose was detected between groups (Figure 4G). We tested insulin sensitivity in peripheral tissues via ITT in vivo and found no difference between male TSC2-KO^Placenta^ and littermate controls (Figure 4H). Moreover, the homeostatic model assessment of insulin resistance (HOMA-IR) at 6 weeks of HFD was comparable between TSC2-KO^Placenta^ and littermate controls (Figure 4I). Together, these data show that adult male TSC2-KO^Placenta^ mice display improved glucose tolerance in HFD.

### Adult male TSC2-KO^Placenta^ mice display hypoinsulinemia and maintain β cell function under HFD challenge.

Insulin resistance is associated with hyperinsulinemia; however, we found significant hypoinsulinemia at week 4 of HFD in male TSC2-KO^Placenta^ compared with littermate controls (Figure 4J). Pancreatic β cell function was assessed in vivo, and GSIS revealed a significant increase in serum insulin secretion only in TSC2-KO^Placenta^ mice under HFD treatment (Figure 4K). Concurrent glucose measurements during the GSIS experiment showed improved glucose tolerance in TSC2-KO^Placenta^ compared with littermate controls (Figure 4L). Pancreatic β cell mass was comparable between TSC2-KO^Placenta^ littermate controls at 10 weeks of HFD (Figure 4M). These studies suggest that TSC2-KO^Placenta^ males were protected from the effects of HFD on glucose tolerance by maintenance of β cell function.

### Gain of placental mTORC1 signaling confers protection from weight gain and HFD-induced glucose intolerance and insulin resistance in female mice.

**Figure 5:**
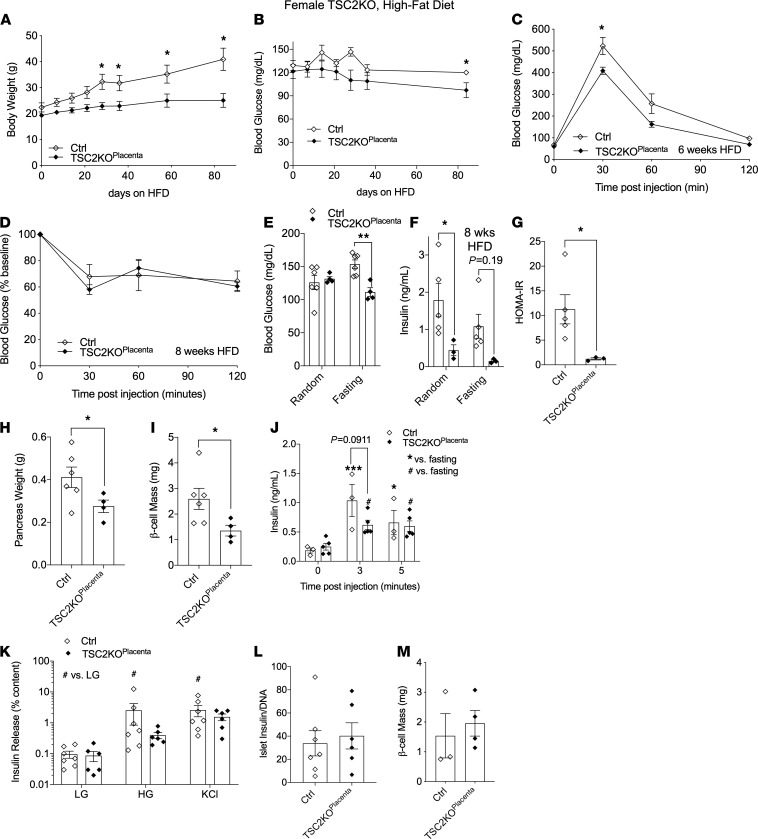
Adult female TSC2-KO^Placenta^ offspring are protected from obesity-induced metabolic dysfunction. Weight progression of female mice fed with HFD over the course of 12 weeks starting at 8 weeks old (*n* = 6 Ctrl, 4 TSC2-KO^Placenta^, **A**). Random blood glucose of HFD females over the duration of treatment (*n* = 6, 4, **B**). Female IPGTT after 16-hour fast at 6 weeks of HFD (*n* = 6, 4, **C**). Female ITT after 6-hour fast at 8 weeks of HFD (*n* = 6, 4, **D**). Blood glucose and insulin serum measurements at random and fasting times of 8-week HFD females (*n* = 6, 4, **E**; *n* = 5, 3, **F**). HOMA-IR of HFD females at 8 weeks (*n* = 5, 3, **G**). Pancreas weight (*n* = 6, 4, **H**) and β cell mass (*n* = 6, 4, **I**) of females at 12 weeks of HFD. In vivo GSIS of aged Ctrl versus TSC2-KO^Placenta^ females under normal diet (*n* = 3, 5, **J**). In vitro GSIS of 12-week-old female islets under low glucose, high glucose, high glucose plus palmitate, and KCl stimulation (*n* = 7, 6, **K**). Insulin content normalized to DNA (*n* = 7, 6, **L**). β Cell mass of female Ctrl versus TSC2-KO^Placenta^ under normal chow (*n* = 3, 4, **M**). Statistical analysis was performed using 2-tailed Mann-Whitney (**G**–**I**, **L**, and **M**) and 2-way ANOVA with Sidak’s multiple comparisons in (**A**–**F**, **J**, and **K**) with repeated measures when appropriate. Error bars represent mean ± SEM. *P < 0.05, **P < 0.01, ***P < 0.001 Ctrl vs. TSC2-KO^Placenta^. ^#^*P* < 0.05 vs. time point 0 or LG (within genotype).

Next we challenged adult female TSC2-KO^Placenta^ and littermate controls to HFD. Unlike the male cohort, female TSC2-KO^Placenta^ mice demonstrated protection from body weight gain throughout the HFD (Figure 5A) associated with lower random blood glucose levels at the end of the treatment (Figure 5B). Female TSC2-KO^Placenta^ mice demonstrated improved glucose tolerance (Figure 5C) but normal insulin sensitivity compared with control littermates at 6 or 8 weeks of HFD, respectively (Figure 5D). At 8 weeks of HFD, nonfasting glucose levels were comparable between TSC2-KO^Placenta^ and littermate controls. However, fasting glucose levels were significantly reduced in TSC2-KO^Placenta^ mice compared with littermate controls (Figure 5E).

### Adult female TSC2-KO^Placenta^ mice display hypoinsulinemia in HFD-induced obesity.

Nonfasting and fasting insulin levels at 8 weeks of HFD were also significantly reduced in TSC2-KO^Placenta^ mice compared with controls, suggesting lack of hyperinsulinemia development in these animals under HFD treatment (Figure 5F). Reduced HOMA-IR index also suggested the presence of increased insulin sensitivity in the TSC2-KO^Placenta^ mice compared with littermate controls (Figure 5G). Together these data suggest that increased placental mTORC1 activity conferred protection from weight gain and HFD-induced glucose and insulin intolerance.

### Adult female TSC2-KO^Placenta^ mice display normal β cell mass but reduced function in control chow diet.

Next, we aimed to identify the contribution of the pancreatic β cell in the improved glucose and insulin tolerance in female TSC2-KO^Placenta^ mice. Pancreatic weight and β cell mass were reduced in TSC2-KO^Placenta^ females at 12 weeks post-HFD compared with controls (Figure 5, H and I). Next, we assessed β cell function in vivo and found that insulin secretion after glucose injection at 3 or 5 minutes reached significance relative to time 0 in both the TSC2-KO^Placenta^ and littermate controls in NCD (Figure 5J). At time point 3 minutes after glucose injection, however, there was a trend toward reduced insulin secretion in female TSC2-KO^Placenta^ compared with control mice (*P* = 0.09, Figure 5J). We then isolated primary islets from TSC2-KO^Placenta^ and control female mice in NCD and performed in vitro GSIS. Insulin secretion in response to high glucose did not reach statistical significance in TSC2-KO^Placenta^ mice compared with littermate controls (Figure 5K). Insulin secretion in response to KCl treatment led to comparable insulin output in both the TSC2-KO^Placenta^ and littermate controls (Figure 5K). Additionally, islet insulin content (Figure 5L) and β cell mass were comparable between TSC2-KO^Placenta^ and littermate controls in NCD (Figure 5M). Together, these data suggest a reduction in β cell function in female offspring with TSC2-KO^Placenta^.

## Discussion

While placental mTOR signaling has been associated with changes in fetal growth in clinical studies, the impact of its activity on long-term metabolic health trajectory in the offspring has not been directly interrogated. Using loss- and gain-of-function mouse models, we demonstrated the impact of placental mTOR signaling on long-term metabolic health of the offspring. Using genetic models, we uncovered that offspring with loss of placental mTOR in utero displayed heightened metabolic dysfunction in response to an HFD challenge. On the other hand, enhanced placental mTORC1 in utero conferred protection from adverse metabolic dysfunction induced by HFD. With loss or gain of mTOR functional studies, we show that placental mTORC1 plays a major role in fetal programming of metabolic responses in the adult offspring, thereby contributing to susceptibility risk for obesity and T2D.

### Placental mTOR signaling’s impact on placental weight and birth weight of newborns.

Enhanced placental mTOR activity has been reported in the placenta of bigger babies born to women with obesity or GDM during pregnancy (27–29). We demonstrated that disruption of mTOR (both mTORC1 and mTORC2) in the placenta resulted in smaller placental weight, whereas enhancing mTORC1 activity did not increase placental weight. Normal body weight was also observed in the offspring with increased placental mTORC1. In agreement with these data pertaining to enhanced mTORC1 activity, maternal protein supplementation that increases mTORC1 does not appear to influence birth weight (30). Increased mTORC1 activity has been correlated with higher birth weight of babies born to women with obesity (31) and GDM (28). However, it is important to note that maternal obesity or GDM has myriad effects, including but not limited to increased maternal glucose, insulin, adipokines, lipids, and low-grade metabolic inflammation, and thus, is not specific to increased mTORC1 activity per se.

### Deficiency in placental mTOR in utero exacerbates responses to adverse metabolic challenge.

The impact of fetal programming is often seen after an adverse exposure, or a “second hit,” encountered later in life. Our data revealed that deficiency in placental mTOR in utero shaped the adult female, but not the male, offspring’s adverse responses to HFD-induced obesity. Male mTOR-KO^Placenta^ and littermate controls responded equally in HFD-induced obesity (data not shown). On the other hand, female mTOR-KO^Placenta^ mice displayed a greater body weight gain throughout the course of HFD, and they displayed exacerbated adiposity and reduced energy expenditure compared with littermate controls. Female mTOR-KO^Placenta^ offspring exposed to HFD also demonstrated worsened glucose intolerance and increased insulin resistance compared with littermate controls. To compensate for increasing insulin demand due to insulin resistance, the pancreas responds by promoting β cell hypersecretion and β cell mass. A comparable β cell mass and a trend toward increase in insulin levels between the female mTOR-KO^Placenta^ and controls suggested insufficient compensation to combat insulin resistance present in the mTOR-KO^Placenta^ offspring. The phenotypes of the mTOR-KO^Placenta^ mice are consistent with rodent females with increased adiposity and disrupted glucose homeostasis because of exposure to in utero undernutrition (26). The current data are also consistent with increased body mass index (BMI), waist circumference, and adiposity and disrupted lipid profile (increased cholesterol and triglycerides) seen in women and not in men (32–36). In maternal undernutrition/FGR in humans, increased BMI and adiposity have been observed in female offspring (32, 33). In rat and mouse models of FGR by maternal low-protein diet during the third trimester of pregnancy, male offspring develop insulin resistance associated with insufficient β cell mass compensation (37, 38). Future studies focused on epigenetic changes in islets may provide mechanistic insights into fetal programming of placental mTOR.

### Increased placental mTORC1 confers protection from adverse metabolic dysfunction induced by obesity.

Limited data suggest that β cell dysfunction persists in small for gestational age infants at 48 hours, but they also tend to be more sensitive to insulin (39). The relationship between insulin secretion and insulin sensitivity is hyperbolic in nature (i.e., insulin secretion decreases in response to greater insulin sensitivity) (40). Adult male and female offspring of dams fed low-protein diet during pregnancy display glucose intolerance despite increased insulin sensitivity in peripheral tissues in part due to impaired β cell dysfunction (4). In contrast, the TSC2-KO^Placenta^ mice appear to have increased insulin sensitivity without glucose intolerance. Under HFD treatment, female TSC2-KO^Placenta^ mice were protected from gaining body weight. These animals displayed improved glucose tolerance and hypoinsulinemia with normal insulin sensitivity (tested by ITT) under HFD. Johnson et al. showed genetic evidence that a reduction of insulin level in circulation prevents HFD obesity (41), and lower circulating insulin enhances insulin sensitivity in mice (42). Lower insulin in circulation also prevents leptin-deficient Lep *ob/ob* mice from developing obesity (43). Therefore, one possible mechanism of metabolic health protection is that lack of hyperinsulinemia contributed to the prevention of weight gain in the TSC2-KO^Placenta^ mice. It will be important for future studies to assess mechanisms of hypoinsulinemia in female TSC2-KO^Placenta^ mice under HFD. In addition to hypoinsulinemia, it will be important to assess the contribution of extrapancreatic tissues by sophisticated clamp studies that can help explain insulin sensitivity in the female TSC2-KO^Placenta^ mice.

### Placental nutrient sensing by mTOR in utero and fetal programming on glucose homeostasis in the adult offspring.

Low–birth weight humans and animals from preclinical models have consistently shown reduced insulin levels, and depending on the timing and severity of the insult, β cell mass reduction can be noted in the offspring across the life span (44). The FGR in the mTOR-KO^Placenta^ was coupled with reduced insulin levels in the offspring serum but normal β cell mass at P0. Increasing placental mTORC1 via TSC2 deletion also did not change basal β cell mass or serum insulin of the offspring at birth. These data are not surprising given that gain of mTORC1 activity (also via deletion of TSC2) directly in β cells does not increase total β cell number in the offspring of dams with normal pregnancy (4); however, it improves β cell mass only in the offspring of low-protein–fed dams (4). The normal glucose homeostasis phenotypes (IPGTT and ITT, summarized in Tables 1 and 2) in male and female adult mTOR-KO^Placenta^ mice further support the lack of changes in β cell mass in the offspring. However, these normal glucose and insulin sensitivity phenotypes under normal diet conditions are in contrast to the observed metabolic outcomes in the offspring of maternal low-protein diet–induced FGR (4) or placental insufficiency models (45–47), thus underscoring the distinct developmental programming effects of specific insults (i.e., placental mTOR vs. maternal low-protein diet or preeclampsia).

Maternal obesity increases placental mTORC1 signaling, and the associated metabolic outcomes in the offspring of obese dams are typically an increased risk for obesity and the metabolic syndrome in adulthood (18, 27). In the current study, gain of placenta-specific mTORC1 signaling in utero led to normal glucose tolerance and enhanced insulin sensitivity phenotypes in male and female offspring under NCD. β Cell mass in males and females was normal. However, adult islets with increased placental mTORC1 in utero showed a significant increase in insulin secretion in response to glucose, but the amount of insulin secreted was markedly less than what was secreted by control islets. Thus, future studies should investigate whether the dampened β cell function phenotype is intrinsic to the pancreas or a response to insulin sensitivity in peripheral tissues.

In the current study, we utilized genetic models with either loss or gain of mTOR signaling in the placenta, and we demonstrated that modulation of placental mTOR impacts long-term metabolic health trajectory of the offspring and that potential augmentation of placental mTORC1 signaling in utero may improve the health of the offspring. We observed sex differences in response to altered placental mTOR signaling, but future work will be required to understand the mechanisms. mTOR regulation of amino acid transporters (12, 25, 48) or mitochondrial biogenesis (49) in the placenta may be involved, but other unbiased analyses are still pending but necessary to fully delineate the mechanisms behind the actions of placental mTOR on fetal programming of key metabolic tissues, such as the pancreas. Abnormal fetal growth affects 20% of all newborn babies around the world and increases their risk for developing obesity, diabetes, and cardiovascular disease in childhood and adulthood (50). To date, no targeted strategies have been established to treat these conditions in utero. The current data presented here advance our understanding of placental mTOR function in pregnancy complications and may provide a foundation for the development of intervention strategies for FGR and fetal overgrowth.

## Methods

### Generation of genetic murine models with loss or gain of placental mTOR function.

Murine models were generated with loss- or gain-of-function mTOR signaling in the placenta. Trophoblast-specific Cre recombinase transgene was used and driven by the CYP19 promoter (20) with *loxP*-flanked sites in either *mTOR* or *TSC2* gene (*mTOR^fl/fl^* or *TSC2^fl/fl^*).

The offspring can be homozygous placental *CYP19Cre^+^ mTOR^fl/fl^* or *CYP19Cre^+^ TSC2^fl/fl^* (mTOR-KO^Placenta^ or TSC2-KO^Placenta^), heterozygous *CYP19Cre^+^ mTOR^fl/WT^* or *CYP19Cre^+^ TSC2^fl/WT^* (mTOR-HET^Placenta^ or TSC2-HET^Placenta^) or *CYP19Cre^–^* with *mTOR^fl/fl^*, *mTOR^fl/WT^*, *TSC2^fl/fl^*, or *TSC2^fl/WT^* (littermate controls). As demonstrated by others, CYP19 was exclusively expressed in the placenta and not in the fetus (20, 22). Ai6 (*CAG-ZsGreen1*) mice (herein referred to as CAG) utilize a CAG system containing ZsGreen1 *loxP*–flanked GFP protein and are used for lineage tracing. Primers used are listed in the key resources table (Supplemental Table 1). The *mTOR^fl/fl^*, *TSC2^fl/fl^*, and Ai6 mice were purchased from The Jackson Laboratory. CYP19Cre mice were donated by G. Leone from Ohio State University. All mice were generated on a mixed background and group housed on a 14-hour light/10-hour dark cycle with ad libitum access to standard diet or HFD (D12492, 60% kcal fat, Research Diets Inc) starting at the age where indicated. For rigor, sex was considered as an independent variable, and data were segregated and analyzed separately.

### Pancreas section collection and β cell mass analysis in animals.

Time pregnancies were set up and observation of a plug were marked as embryonic day 0.5. At E17.5 or E18.5, the dam was sacrificed, and the pancreas was harvested from the fetus. Newborn pancreata were collected at birth (P0). Harvested newborn and embryonic pancreata were soaked in 3.75% formalin for 5 hours, or overnight for adult pancreata, then in 70% ethanol prior to embedding. Adult tissue blocks were sectioned at 5 μm thickness until 50 μm of tissue was collected, then removed 200 μm from the block, repeating until 5 different areas of the pancreas were collected. Newborn or embryonic tissue blocks were collected from top to bottom at 5 μm thickness. Analysis of β cell mass was previously described (51).

### Immunostaining and histology quantification.

Immunofluorescence and IHC techniques were utilized in paraffin-embedded mouse or human tissues as previously described (52). H&E was used to assess macroscopic observation of the mouse placenta. Sagittal cuts were performed on the middle section of murine placenta and paraffin-embedded cut side down. The standard Citri Solv (Thermo Fisher Scientific) deparaffinization and dehydration procedure was done on both mouse and human tissue. Both H&E and IHC staining were performed per manufacturer’s protocol. All murine samples were visualized using Nikon ECLIPSE Ni-E microscope. Quantification of chromogen immunostaining was analyzed using ImageJ (NIH, http://imagej.nih.gov/ij/). See Supplemental Table 1 for key resources information.

### Tissue clearing and microscopy of newborn animals.

The tissue was fixed in fresh 4% paraformaldehyde in 0.1 M PBS, pH 7.2, for 24 hours. Then the fixed tissue was cleared using the Logos Biosystems X-CLARITY System as previously described (53). Images were taken using the Ni-E microscope with C2+si confocal microscope in spectral mode utilizing GaAsP spectral detectors by using the Nikon 10× glycerol, 0.5 NA, 5.5 mm WD objective (Nikon Instruments) while using the 488 nm laser and collecting emission spectra from 500–650 nm at 10 nm spectral resolution. Tiled, *Z*-series images were spectrally unmixed using EGFP library spectra and processed in Nikon Elements v5.01. The tissue clearing and microscopy were performed in the University Imaging Centers, University of Minnesota (https://med.umn.edu/uic).

### Primary mouse islet isolation, Western blot, insulin secretion in vitro, and glucose and insulin levels.

Islets were isolated by collagenase digestion through the common duct as previously described (54). After handpicking the islets and allowing an overnight rest period, the islets were prepped for in vitro glucose stimulus insulin secretion assays as described (54). Protein samples were prepared from 3 to 4 pancreata, livers, and inguinal fat pads from each treatment group. Samples were flash frozen with liquid nitrogen and then homogenized. Samples were washed with 1× PBS prior to incubating in lysis buffer (Cell Signaling Technology) with protease inhibitor and phosphatase inhibitor cocktails (Roche Applied Science). Western blot preparation, procedure, and densitometry analysis were done as previously described (51). See Supplemental Table 1 for primary antibodies used. Glucose (detected by glucose meter) and insulin (assessed by ELISA) were measured from trunk blood collected from P0 animals as previously described (51).

### AA analysis and leucine placental perfusion experiment.

Serum AAs from P0 (collected from trunk blood) were measured by simultaneous liquid chromatography–mass spectrometry (LC-MS) as previously described (55). Briefly, samples were derivatized with dansyl chloride (DC) prior to the LC-MS analysis. A total of 5 μL of sample or standard was mixed with 5 μL of 100 μM *p*-chlorophenylalanine (internal standard), 50 μL of 10 mM sodium carbonate, and 100 μL of DC (3 mg/mL in acetone). The mixture was incubated at 60°C for 15 minutes and centrifuged at 18,000*g* for 10 minutes. The supernatant was transferred into an HPLC vial (Thermo Fisher Scientific) for LC-MS analysis. Unidirectional flow from materno-fetal placental interface was measured using radiolabeled isotopes as previously described (56). At gestation day 17.5, the maternal jugular vein was exposed under isoflurane anesthesia. A total of 100 μCi of ^3^H-Leucine was injected through a jugular catheter. Blood was collected at time points 0 and 10 minutes via tail vein before and after injection, respectively, for count readings to ensure successful injection. After 10 minutes, the dam was euthanized. The placenta and fetus (postdecapitation) were placed in scintillation tubes with Biosol and incubated overnight in a water bath at 95°C for digestion. The resulting lysates were diluted 1:10 with Bioscint and then ^3^H counted with liquid scintillation counter (Beckman Coulter, LS6500).

### Metabolic phenotyping.

Glucose and insulin tolerance tests were performed by intraperitoneal delivery of 2 g/kg glucose or 0.75 U/kg insulin (Novolin, Novo Nordisk Inc) to mice after 12 or 6 hours of fasting as previously described (57). The HOMA-IR index was calculated as (fasting insulin [ng/mL] × fasting glucose [mmol/L])/22.5 to assess insulin resistance. Metabolic cages for whole-animal energy expenditure (Oxymax/CLAMS Lab Animal Monitoring System, Columbus Instruments) and body composition via EchoMRI (Echo Medical Systems LLC) service were provided by the University of Minnesota Integrative Biology and Physiology Core.

### Statistics.

Data were analyzed by using Mann-Whitney *t* test (2 tailed) or 2-way ANOVA with Sidak’s multiple comparisons test as indicated in the legends. A *P* value of less than 0.05 was considered statistically significant. Data were evaluated by GraphPad Prism 7 or 8 (GraphPad Software).

### Study approval.

All animal studies were approved by the Institutional Animal Care and Use Committee (protocol 1806-36072A) at the University of Minnesota, and radiation studies were approved by the University Health and Safety Department of Radiation Safety at the University of Minnesota.

## Author contributions

BA, DCB, SJ, ADL, MB, AW, TZ, RM, MS, DLF, and EUA designed experiments, generated and analyzed data, assisted with manuscript preparation, and approved the final version; BA and EUA interpreted the data and wrote and edited the manuscript; MRBPC contributed to the discussion; and EUA conceived the study, acquired funding, was in charge of overall direction of this work, and is the guarantor of this work.

## Supplementary Material

Supplemental data

## Figures and Tables

**Table 1:**
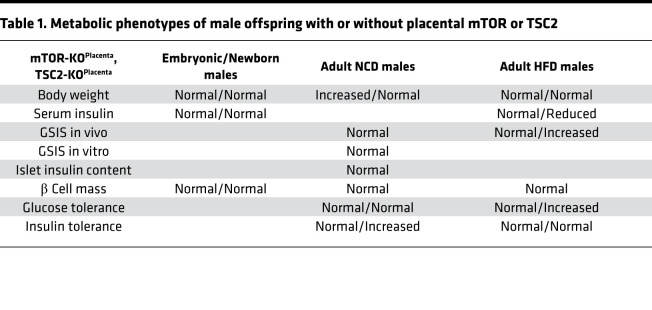
Metabolic phenotypes of male offspring with or without placental mTOR or TSC2

**Table 2:**
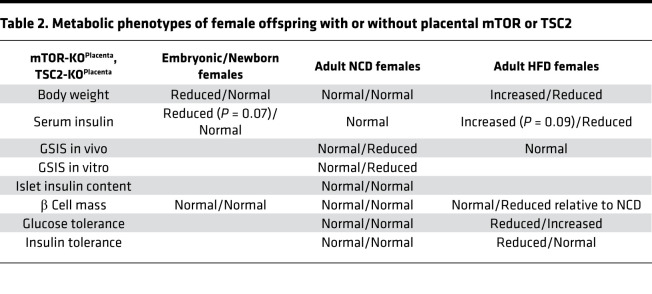
Metabolic phenotypes of female offspring with or without placental mTOR or TSC2

## Version 1 05/25/2021

In-Press Preview

## Version 2 07/08/2021

Electronic publication
