# Supplementary material for: Placental mTOR complex 1 regulates fetal programming of obesity and insulin resistance in mice
Source: JCI Insight. 2021 Jul 8;6(13):e149271. doi: 10.1172/jci.insight.149271 (PMC8410096; doi:10.1172/jci.insight.149271)
Supplement: Supplemental data [file jciinsight-6-149271-s082.pdf]

S. Figure 1

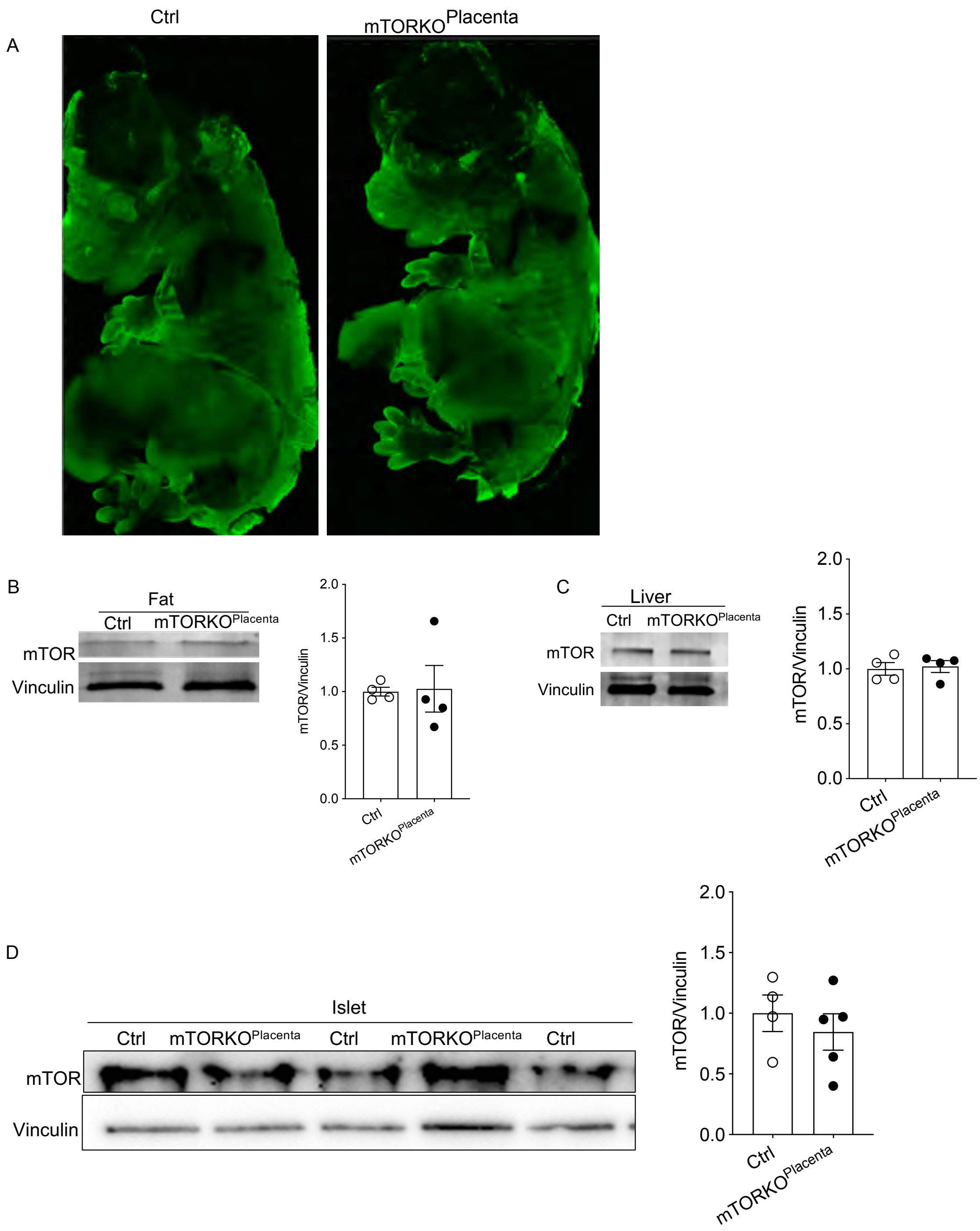
A
Ctrl
mTORKO Placenta
B
Fat
Ctrl
mTORKO Placenta
mTOR
Vinculin
mTOR/Vinculin
2.0
1.5
1.0
0.5
0.0
Ctrl
mTORKO Placenta
C
Liver
Ctrl
mTORKO Placenta
mTOR
Vinculin
mTOR/Vinculin
2.0
1.5
1.0
0.5
0.0
Ctrl
mTORKO Placenta
D
Islet
Ctrl
mTORKO Placenta
Ctrl
mTORKO Placenta
Ctrl
mTOR
Vinculin
mTOR/Vinculin
2.0
1.5
1.0
0.5
0.0
Ctrl
mTORKO Placenta

S. Figure 2

Male and Female mTORKO Offspring

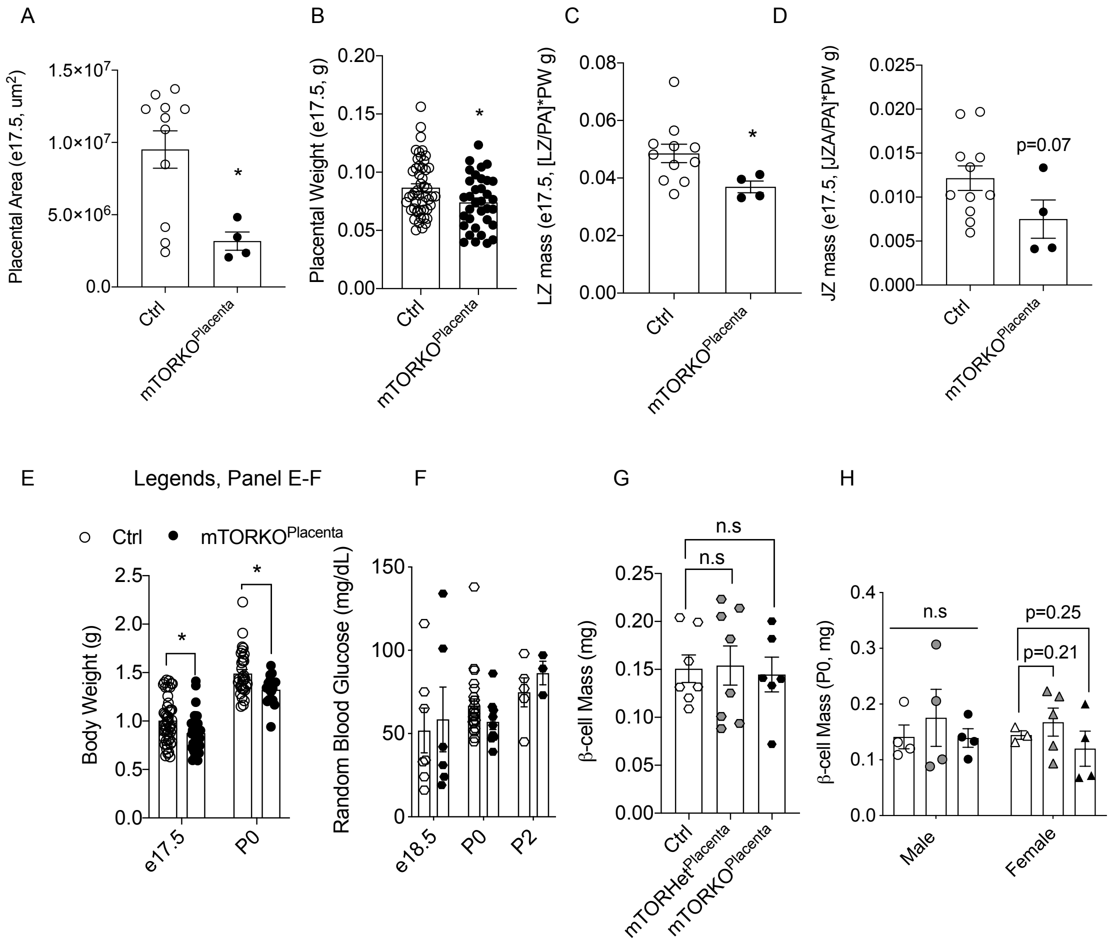

S. Figure 3

A

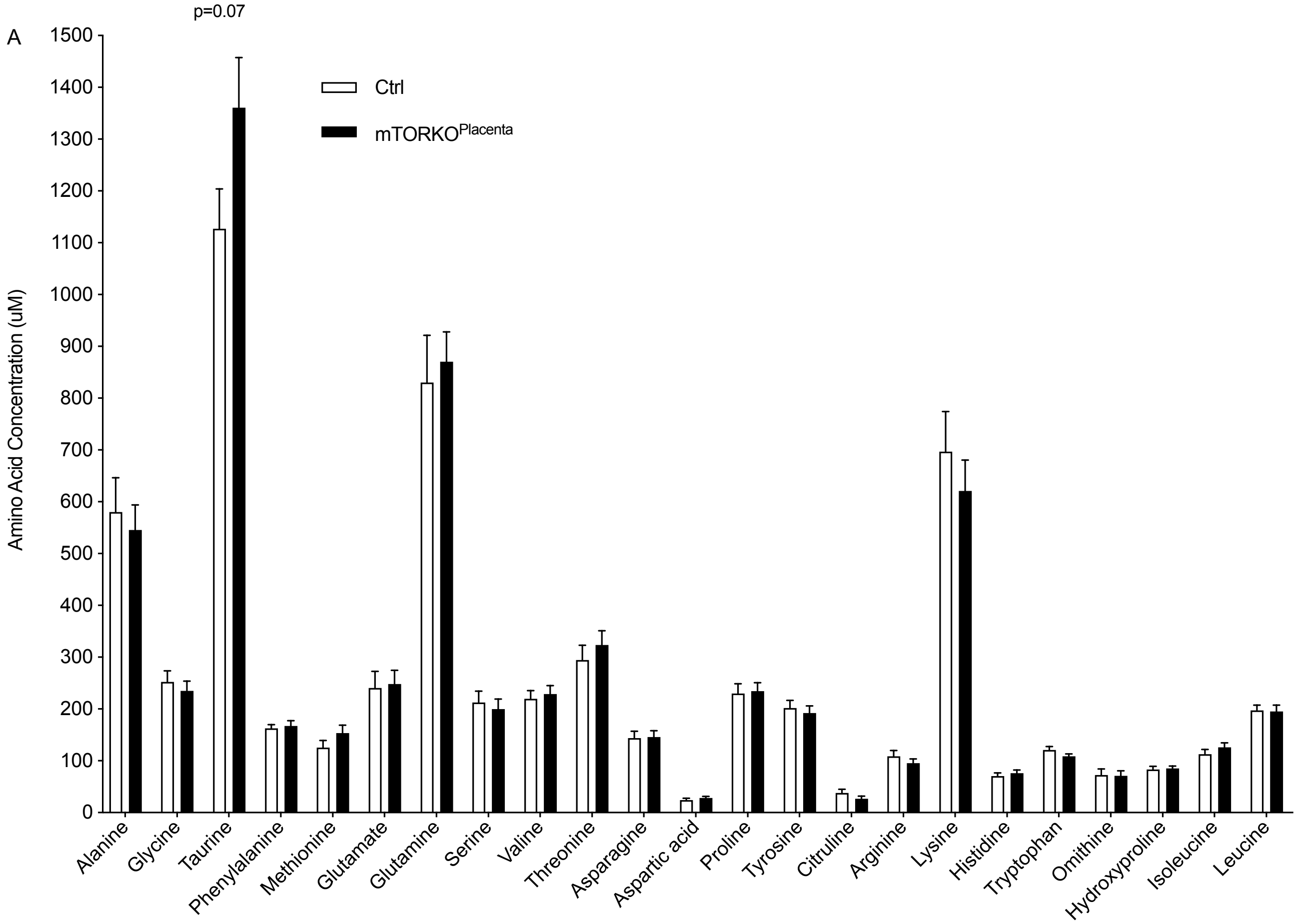

p=0.07
1500
1400
1300
1200
1100
1000
900
800
700
600
500
400
300
200
100
0
Amino Acid Concentration (uM)
Ctrl
mTORKO^Placenta
Alanine
Glycine
Taurine
Phenylalanine
Methionine
Glutamate
Glutamine
Serine
Valine
Threonine
Asparagine
Aspartic acid
Proline
Tyrosine
Citruline
Arginine
Lysine
Histidine
Tryptophan
Ornithine
Hydroxyproline
Isoleucine
Leucine

S. Figure 4

Male and Female mTORKO, Normal Chow Diet

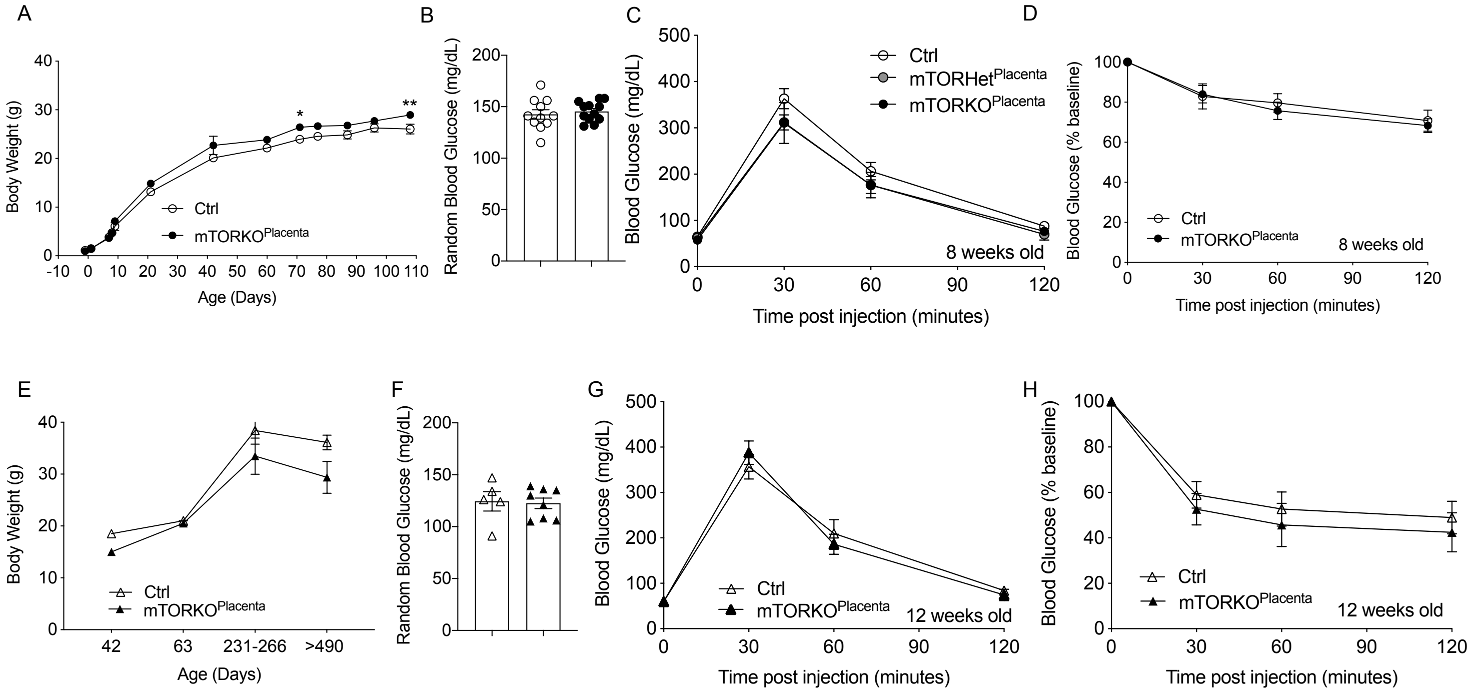

S. Figure 5

Male and Female TSC2KO Offspring

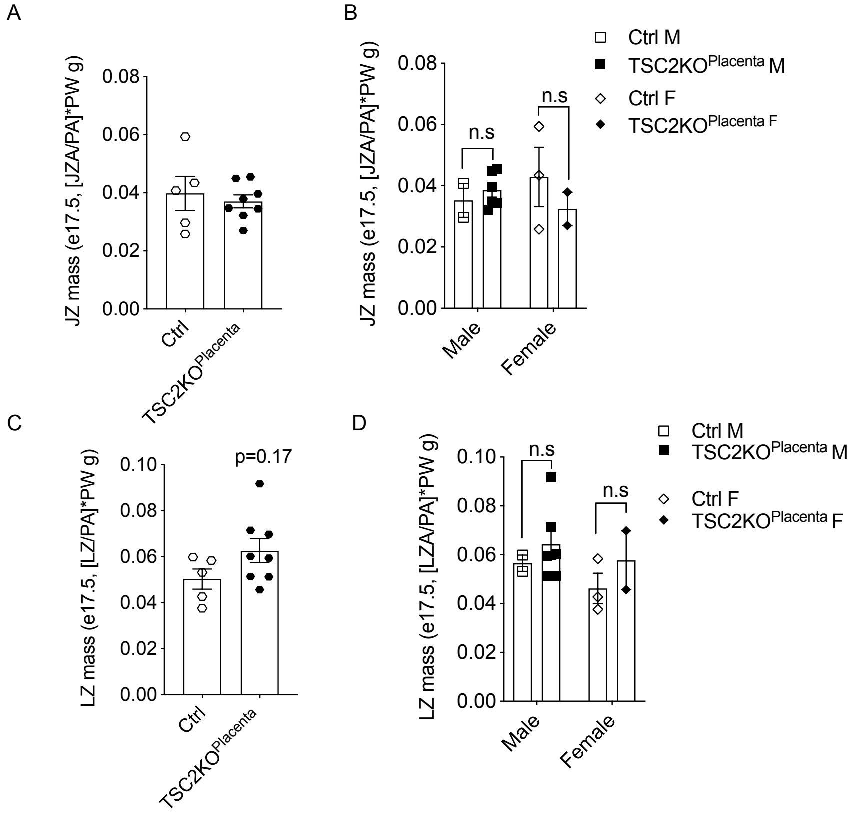

S. Figure 6

Male and Female TSC2KO, Normal Chow Diet

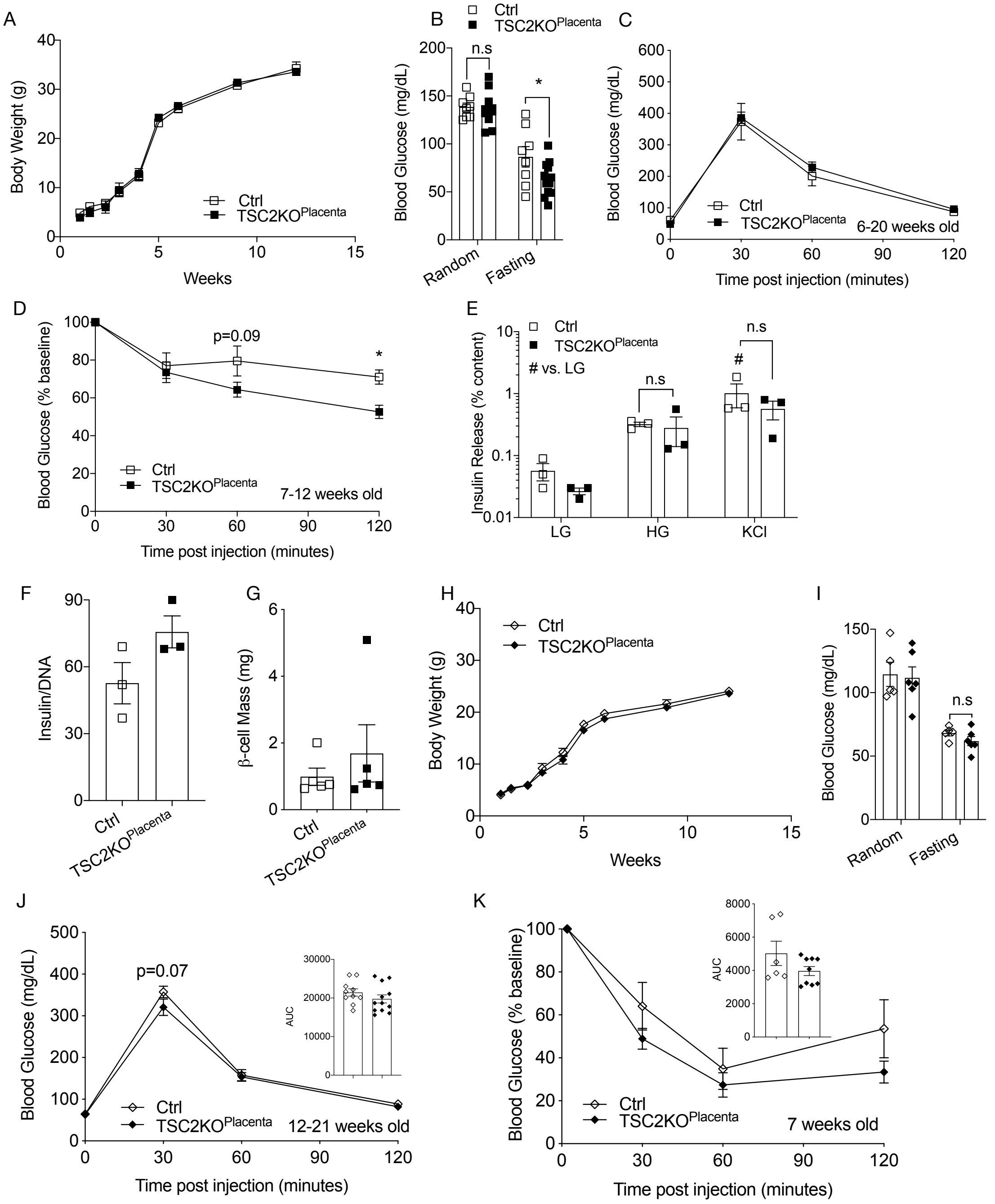

Supplemental Legends:

S. Figure 1. *Validation of CYP19Cre model and mTOR protein levels in key metabolic tissues in the offspring.* Images of embryonic pancreas using immunofluorescence imaging and whole embryos using x-clarity tissue clearing system (**A**). Representative western blot of WAT (white adipose tissue) for mTOR and quantification normalized to vinculin as loading control (**B**). Representative western blot of liver lysates for mTOR and quantification normalized to vinculin (**C**). Representative western blot of isolated islets for mTOR with respective quantifications normalized to vinculin (**D**). Statistical analysis was performed using two-tailed Mann-Whitney t-test **(B-D)**. Error bars are represented by ± SEM. Error bars are represented by ± SEM. $^*p < 0.05$.

S. Figure 2. *Consolidated weights, random blood glucose, random insulin, and β-cell mass of mTORKO^{Placenta} animals.* Combined placental area (n=11,4, **A**) and weight (n=11,4, **B**) of e17.5 neonates. Labyrinth zone (LZ) mass of e17.5 placentas (n=11,4, **C**). Junctional zone (JZ) mass of e17.5 placentas (n=11,4, **D**). Ctrl vs. mTORKO^{Placenta} fetal weight of e17.5 (n=29,16, **E**) and post-natal day 0 (P0) pups (n=23,8, **E**). Random (non-fasted) blood glucose from the trunk of e18.5 (n=7,6), P0 (n=25,9), and P2 pups (n=5,3, **F**). β-cell mass in P0 newborn animals (**G**). β-cell mass of P0 control, mTORHET^{Placenta} and mTORKO^{Placenta} males (n=14,5,11) and females (n=10,4,5, **H**). Statistical analysis was performed using two-tailed Mann-Whitney t-test (**A-D**), two-way ANOVA Sidak's multiple comparisons (**E**, **F**, **H**), and one-way ANOVA Dunnett's multiple comparison (**G**). Error bars are represented by ± SEM. $^*p < 0.05$.

S. Figure 3. *Comparable amino acids levels in mTORKO^{Placenta} and control offspring*

A. No changes in AA detected between mTORKO^{Placenta} and control offspring at P0. Statistical analysis was performed using two-way ANOVA Sidak's multiple comparisons. Error bars are represented by ± SEM. *p< 0.05.

S. Figure 4. *Adult male and female mTORKO^{Placenta} offspring display normal glucose homeostasis under normal chow diet*. Body weight over time of male offspring from birth to 110 days of age in normal chow diet (NCD) (at least n=5, **A**). Random blood glucose of 12-20 weeks old Ctrl and mTORKO^{Placenta} males (n=11,12, **B**). IPGTT (n=8,3,6) and ITT (n=6) of 8 week old male mice in NCD (**C**-**D**). Body weight of Ctrl vs. mTORKO^{Placenta} females over 6, 9, 33-48, and 70 weeks of age in NCD (**E**). Random blood glucose of 11-21 weeks old females in NCD (n=5,8, **F**). IPGTT on 12 weeks old mTOR Ctrl and mTORKO^{Placenta} females in NCD (n=10,6, **G**). ITT blood glucose percentile of 12 weeks old female in NCD (n=4, **H**). Statistical analysis was performed using two-tailed Mann-Whitney t-test (**B**, **F**) or two-way ANOVA with Sidak's multiple comparisons and repeated measures (**A**, **C**-**E**, **G**, **H**). *p< 0.05, ** p< 0.01. Error bars are represented by ± SEM.

S. Figure 5. *Placental phenotype of TSC2KO^{Placenta} male and female animals*. Placental JZ mass in e17.5 Ctrl and TSC2KO^{Placenta} (n=5,8, **A**) and separated by sex (**B,** male (n=2,6) and female (n=3,2). Combined LZ mass in e17.5 Ctrl and TSC2KO^{Placenta} (n=5,8, **C**) and separated by sex (**D,** male (n=2,6) and female (n=3,2). Statistical analysis was performed using two-tailed Mann-Whitney t-test (**A**, **C**) or two-way ANOVA with Sidak's multiple comparisons test (**B**, **D**). *p< 0.05, ** p< 0.01. Error bars are represented by ± SEM.

S. Figure 6. *Adult male TSC2KO^{Placenta} offspring have improved insulin tolerance in normal chow diet.* Progression of male Ctrl vs. TSC2KO^{Placenta} body weight over time from 1-12 weeks of age

under NCD (**A**). Random and fasting blood glucose of 4-7 weeks old males (n=8-10, **B**). IPGTT of 6-20 weeks old males (n=6,12, **C**). ITT of 7-12 weeks old males (n=6,8, **D**). *In vitro* GSIS of 12-36 weeks old male islets under low glucose, high glucose, high glucose plus palmitate, and KCl stimulation (n=3, **E**). Islet insulin content normalized to DNA (n=3, **F**). β-cell mass of male Ctrl vs. $TSC2KO^{Placenta}$ under normal chow (n=5, **G**). Weight progression of Ctrl vs. $TSC2KO^{Placenta}$ female mice under normal chow (**H**). Random and fasting blood glucose of Ctrl vs. $TSC2KO^{Placenta}$ female mice under normal chow (n=5,6, **I**). IPGTT on 12-21 weeks old females (n=10,13) and AUC (**J**). ITT on 7 weeks old females (n=6,9) and AUC (**K**). Statistical analysis was performed using two-tailed Mann-Whitney t-test (**F**, **G**) or two-way ANOVA with Sidak's multiple comparisons test (**A**-**E**, **H**-**K**) and repeated measures when appropriate. Error bars are represented by ± SEM. $*p < 0.05$.

S. Table 1: Key resources information.

S. Table 1: Key Resources Table

REAGENT or RESOURCE	SOURCE	IDENTIFIER
**Antibodies**		
Insulin Antibody, guinea pig polyclonal (1:400)	Dako	Cat# A056401-2, RRID: AB2617169
mTOR (7C10) Antibody, rabbit monoclonal (1:400)	Cell Signaling Technology	Cat# 2983S, RRID: AB2105622
Phospho-S6 (Ser235/236), rabbit monoclonal (1:400)	Cell Signaling Technology	Cat# 4858S, RRID: AB916156
Phospho-S6 (Ser240/244), rabbit monoclonal (1:400)	Cell Signaling Technology	Cat# 5364S, RRID: AB916156
Beta-Actin (8H10D10) Antibody, mouse monoclonal (WB 1:1000-2000)	Cell Signaling Technology	Cat# 3700, RRID: AB2242334
Vinculin (E1E9V) Antibody, rabbit monoclonal (WB 1:1000-2000)	Cell Signaling Technology	Cat# 13901S, RRID: AB2728768
Sheep Anti-Mouse IgG (HRP-conjugate) (WB 1:10,000)	GE Healthcare	Cat# NA931, RRID: AB772210
Donkey Anti-Rabbit IgG (HRP-conjugate) (WB 1:15,000)	GE Healthcare	Cat# NA934, RRID: AB772206
Biotinylated anti-mouse (IHC 1:500)	Vector Laboratories	BA-9200-1.5
Biotinylated anti-rabbit (IHC 1:500)	Vector Laboratories	BA-1000-1.5
IRDYE 680RD Goat Anti-Rabbit (WB 1:10000)	Li-Cor	925-68071
**Chemicals, Peptides, and Recombinant Proteins**		
RPMI 1640 Media	Corning	10-043-CV
HBSS (+ or - Ca2+, Mg2+)	Gibco	14025-092 (+), 14175-095 (-)
Fetal Bovine Serum	GenClone	25-514
RIPA Buffer	Cell Signaling Technology	9806S

Protease Inhibitor Cocktail	Cell Signaling Technology	5871S
Phosphatase Inhibitor Cocktail	Cell Signaling Technology	5870S
Collagenase P	Sigma-Aldrich (Roche)	11213865001
Pierce BCA Protein Assay Kit	ThermoFisher Scientific	23227
RNeasy Mini Kit	Qiagen	74104
Rodent Diet with 60% kcal fat	Research Diets	D12492 (HFD)
Humalog Insulin Lispro, injectable solution (100 iU/mL)	Eli Lilly and Company	NDC 0002-7510-01
50% Dextrose Injection, USP	Hospira, Inc.	NDC 0409-6648-02, RL-3040
DAPI mounting media	Vector Laboratories	H-1200
Palmitic Acid	Fisher (Nu-chek Prep, Inc)	NC0841173
Albumin, Bovine Serum, Fraction V, RIA and ELISA Grade	Millipore Sigma	126593
RNAlater	ThermoFisher Scientific	AM7024
DMSO	Sigma-Aldrich	D2650
RestoreTM PLUS Western Blot Stripping Buffer	ThermoFisher Scientific	46430
Odyssey Blocking Buffer (TBS)	Li-Cor	927-50000
Biosol	National Diagnostic	LS-310
Bioscint	National Diagnostic	LS-309
Hematoxylin	Abcam	ab220365
Eosin Y	Sigma Aldrich	HT110116

Formula 83	ThermoFisher Scientific	CH0104A
Rabbit Specific HRP/DAB (ABC) Detection IHC Kit	Abcam	ab64261
**Critical Commercial Assays**		
Mouse Ultrasensitive Insulin ELISA	ALPCO	80-INSMU-E01
Contour Blood Glucose Meter and Test Strips	Bayer	9545C (meter), 06707202 (strips)
Quant-iT PicoGreen dsDNA Assay Kit	ThermoFisher Scientific	Ref: P11496
Pierce BCA Protein Assay Kit	ThermoFisher Scientific	23227
SuperSignal West Pico PLUS Chemi	ThermoFisher Scientific	34580
Primer: CYP19cre Forward: GAC CTT GCT GAG ATT AGA TC	IDT, This Paper	N/A
Primer: CYP19cre Reverse: GAC GAT GAA GCA TGT TTA GCT GGC C	IDT, This Paper	N/A
Primer: mTOR floxed Reverse: TTT AGG ACT CCT TCT GTG ACA TAC ATT TCC T	IDT, This Paper	N/A
Primer: mTOR Forward: TTA TGT TTG ATA ATT GCA GTT TTG GCT AGC AGT	IDT, This Paper	N/A
Primer: TSC2 floxed reverse: AAG CAG CAG GTC TGC AGT G	IDT, This Paper	N/A
Primer: TSC2 floxed Forward: ACA ATG GGA GGC ACA TTA CC	IDT, This Paper	N/A
Isotopes		
L-[4,5-^{3}H(N)]-Leucine	Perkin Elmer	NET135H001MC
α-[1-^{14}C]-Methylaminoisobutyric Acid	Perkin Elmer	NEC671250UC
Software and Algorithms		
ImageJ	https://imagej.nih.gov/ij/	RRID:SCR_003070

Prism v.7.0d	https://www.graphpad.com/scientific-software/prism/	RRID:SCR_002798
